# Resistance to immune checkpoint inhibitors in advanced lung cancer: Clinical characteristics, potential prognostic factors and next strategy

**DOI:** 10.3389/fimmu.2023.1089026

**Published:** 2023-01-26

**Authors:** Jiebai Zhou, Xinyuan Lu, Haixing Zhu, Ning Ding, Yong Zhang, Xiaobo Xu, Lei Gao, Jian Zhou, Yuanlin Song, Jie Hu

**Affiliations:** ^1^ Department of Pulmonary and Critical Care Medicine, Zhongshan Hospital, Fudan University, Shanghai, China; ^2^ Key Laboratory of Public Health Safety, School of Public Health, Ministry of Education, Fudan University, Shanghai, China; ^3^ Department of Pulmonary and Critical Care Medicine, Ruijin Hospital, Shanghai Jiao Tong University School of Medicine, Shanghai, China; ^4^ Shanghai Key Laboratory of Lung Inflammation and Injury, Shanghai, China; ^5^ Department of Pulmonary and Critical Care Medicine, Shanghai Geriatrics Center, Shanghai, China

**Keywords:** immune checkpoint inhibitor, resistance, advanced lung cancer, plasma cytokines, prognostic factor

## Abstract

**Background:**

Immune checkpoint inhibitors (ICIs) have shown unprecedented clinical benefit in cancer immunotherapy and are rapidly transforming the practice of advanced lung cancer. However, resistance routinely develops in patients treated with ICIs. We conducted this retrospective study to provide an overview on clinical characteristics of ICI resistance, optimal treatment beyond disease progression after prior exposure to immunotherapy, as well as potential prognostic factors of such resistance.

**Methods:**

190 patients diagnosed with unresectable lung cancer who received at least one administration of an anti-programmed cell death 1 (PD-1)/anti-programmed cell death-ligand 1(PD-L1) at any treatment line at Zhongshan Hospital Fudan University between Sep 2017 and December 2019 were enrolled in our study. Overall survival (OS) and progression-free survival (PFS) were analyzed. Levels of plasma cytokines were evaluated for the prognostic value of ICI resistance.

**Results:**

We found that EGFR/ALK/ROS1 mutation and receiving ICI treatment as second-line therapy were risk factors associated with ICI resistance. Patients with bone metastasis at baseline had a significantly shorter PFS1 time when receiving initial ICI treatment. Whether or not patients with oligo-progression received local treatment seemed to have no significant effect on PFS2 time. Systemic therapies including chemotherapy and anti-angiogenic therapy rather than continued immunotherapy beyond ICI resistance had significant effect on PFS2 time. TNF, IL-6 and IL-8 were significantly elevated when ICI resistance. Lower plasma TNF level and higher plasma IL-8 level seemed to be significantly associated with ICI resistance. A nomogram was established to prognosis the clinical outcome of patients treated with ICIs.

**Conclusion:**

Patients with EGFR/ALK/ROS1 mutation, or those receiving ICI treatment as second-line therapy had higher risk of ICI resistance. Patients with bone metastasis had poor prognosis during immunotherapy. For those patients with oligo-progression after ICI resistance, combination with local treatment did not lead to a significantly longer PFS2 time. Chemotherapy and anti-angiogenic therapy rather than continued immunotherapy beyond ICI resistance had significant effect on PFS2 time. Levels of plasma cytokines including TNF, IL-6 and IL-8 were associated with ICI resistance.

## Introduction

The advent of immune checkpoint inhibitors (ICIs) has made an indelible mark in the field of cancer immunotherapy. ICIs promote recognition of cancer cells as foreign cells by the immune system and reverse the tumor-driven inhibition of the immune system that promotes tumor growth (1, 2). ICIs targeting the programmed cell death 1 (PD-1)/programmed cell death-ligand 1 (PD-L1) axis have altered the treatment landscape for patients with lung cancer. They have been implemented in the clinical routine as a standard of care, either as monotherapy or in combination with chemotherapy in advanced non-oncogene-driven non-small cell lung cancer (NSCLC), in combination with chemotherapy in extensive stage small cell lung cancer (SCLC) and as a consolidation therapy in unresectable stage III NSCLC.

However, responses to ICI therapy are not ubiquitous. It has been observed that a proportion of patients do not respond initially to ICI, displaying primary, also known as innate, resistance to ICI therapy. In addition, a number of patients who derive an initial clinical benefit from ICI will subsequently relapse, exhibiting secondary or acquired resistance (3). Primary resistance to immunotherapy accounts for 7–27% of first-line treatment and 20–44% of second-line treatment in patients with lung cancer (4). Approximately 25% of patients treated with ICIs could develop acquired resistance (5). This encourages the identification of resistance mechanisms associated with immunotherapy in the hope of preventing them and developing strategies to overcome them, as well as the development of new and more effective strategies to increase the number of patients who can benefit from immunotherapy.

Considering the limited data in regards of management of resistance to ICI in lung cancer, we planned a retrospective study with the aim of demonstrating clinical characteristics of ICI resistance, proposing optimal treatment approaches in patients who have progressed after prior exposure to immunotherapy and providing an overview on potential prognostic factors of such resistance.

## Material and methods

### Study subjects and study approval

The medical records of 199 consecutive patients with lung cancer who received at least one administration of an anti-PD1/anti-PD-L1 ICI at any treatment line at Zhongshan Hospital Fudan University between Sep 2017 and December 2019 were retrospectively reviewed. Data were collected from our electronic medical record and patient follow-up visits. We excluded 9 patients for diagnosis of resectable lung cancer. Finally, 190 evaluable patients were included.

This study was approved by the ethics committee of Zhongshan Hospital, Fudan University (No. B2020-431R). The requirement for informed consent from the patients included in this study was waived due to the retrospective nature of the study, and any personal information from the data was removed beforehand. The censored date was June 29, 2021.

### Data collection

The data collected for all patients included gender, age, smoking history, pack-years index, medical history, performance status (PS) on the Eastern Cooperative Oncology Group scale, tumor-node-metastasis (TNM) staging, histology, driven mutation, PD-L1 expression, treatment regimen, toxicity profile, and survival.

### Evaluation of responses and toxicity

We evaluated the overall survival (OS) and progression-free survival (PFS). OS was measured as the period from the diagnosis of lung cancer to death. PFS1 was measured as the period from the first administration of immunotherapy to documented disease progression or death from any cause. PFS2 was measured as the period from the initiation of systemic treatment beyond disease progression after the initial immunotherapy to documented second progression or death from any cause. Treatment response was assessed using the Response Evaluation Criteria in Solid Tumors version 1.1. Adverse events were graded using the National Cancer Institute Common Terminology Criteria for Adverse Events (version 4.0).

### Measurement of cytokines

Cytokines were detected by a solid-phase, two-site chemiluminescent immunometric assay.

### Statistical analysis

Numbers and percentages were computed for categorical variables. Means and standard deviations for normally distributed continuous variables and medians and interquartile ranges for abnormally distributed continuous variables were calculated. The cumulative rates of OS or PFS were estimated with Kaplan-Meier method and survival curves were plotted. Comparisons between survival curves were performed by log-rank tests.

Factors affecting OS or PFS were analyzed using Cox proportional hazard model. Factors with statistical significance in univariate analysis were then put into multivariate regression models to further identify their effects on fatal or progressive outcomes. Hazard ratios (HR), together with its 95% confidence intervals were reported. Forest plots were drawn to visualize the results of multivariate analysis.

Comparisons between cytokine levels of TNF, IL-2R, IL-6, IL-8 and CD4/CD8 at diverse detection time and baseline levels were performed using Wilcoxon test due to abnormal distributed data. In order to build a predictive model for PFS with cytokine levels, cutoff values were determined using median values or receiver operating characteristics curves analysis. If area under curve (AUC) was smaller than 0.5, the median value was considered the cutoff value. Otherwise, cutoff values were calculated based on ROC. The results of predictive model were visualized using nomogram whose performance was evaluated by Harrell concordance index (C-index). 1000 bootstrap resamples were used for internal validation of the accuracy of the predictions. Calibration curves were drawn to assess the consistence between the predicted probability and the actual proportion (6). All statistical analyses were performed by R version 4.1.0. A two-sided P value less than 0.05 was considered as the significance level.

## Results

### Demographic information and baseline characteristics of the patients

**Figure 1:**
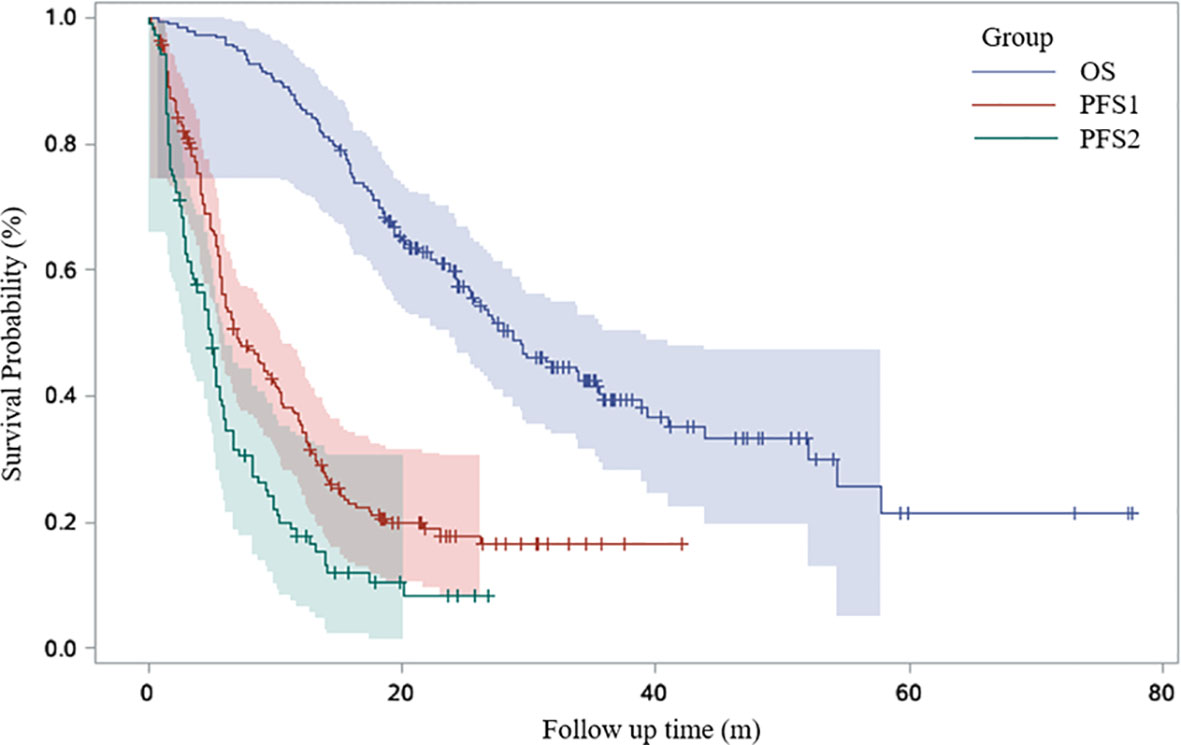
Kaplan-Meier curves for OS, PFS1 and PFS2 of the entire cohort.

Detailed demographic information and baseline characteristics of the studied subjects are presented in **Table 1**. Among the 190 patients enrolled in our cohort, 84 (44.2%) patients received ICI as first line therapy. 73 (38.4%) patients received ICI as monotherapy. The objective response rate (ORR) of immunotherapy in our patients was 36.3%. The Kaplan-Meier curves for all the patients are shown in **Figure 1**. The median OS was 28.7 months. Median PFS1 and PFS2 were 6.9 and 4.8 months, respectively.

**Table 1:** Baseline characteristics of study population.

Characteristics	Total number (N)	Percentage (%)
Gender		
Male	163	85.8
Female	27	14.2
Age		
≤65	118	62.1
>65	72	37.9
Smoking history		
Never	71	37.4
≤40 pack-years	61	32.1
>40 pack-years	58	30.5
Medical history		
Respiratory diseases	25	13.2
Hypertension	64	33.7
Endocrine system diseases	22	11.6
Musculoskeletal diseases	8	4.2
Cardiovascular and cerebrovascular diseases	16	8.4
Digestive diseases	14	7.4
Urogenital diseases	3	1.6
Cancer type		
Adenocarcinoma	78	41.1
Squamous cell lung cancer	50	26.3
Small cell lung cancer	26	13.7
Other types	36	18.9
TNM stage		
II/III	36	18.9
IV	154	81.1
PS		
0	95	50.0
1	74	38.9
≥2	13	6.8
EGFR/ALK/ROS1 mutation		
No	84	44.2
Yes	23	12.1
NO-test	83	43.7
Metastatic information at baseline		
No-metastasis	41	21.6
Oligo-metastasis	86	45.3
Multi-metastasis	63	33.2
Metastatic sites		
Lung	39	20.5
Liver	30	15.8
Bone	53	27.9
Brain	35	18.4
Adrenal gland	25	13.2
Pleura	40	21.1
Other	20	10.5
Therapy		
Monotherapy	73	38.4
Combined chemotherapy	110	57.9
Combined anti-angiogenic agents	7	3.7
Treatment line		
First-line	84	44.2
Second-line	52	27.4
Subsequent line	54	28.4
PD-L1 expression		
Negative	49	25.8
1-49%	48	25.3
≥50%	34	17.9
Best efficacy of immunotherapy		
PD	31	16.3
PR	69	36.3
SD	90	47.4
irAE		
No	150	78.9
Yes	40	23.5

### Risk factors associated with ICI resistance

**Figure 2:**
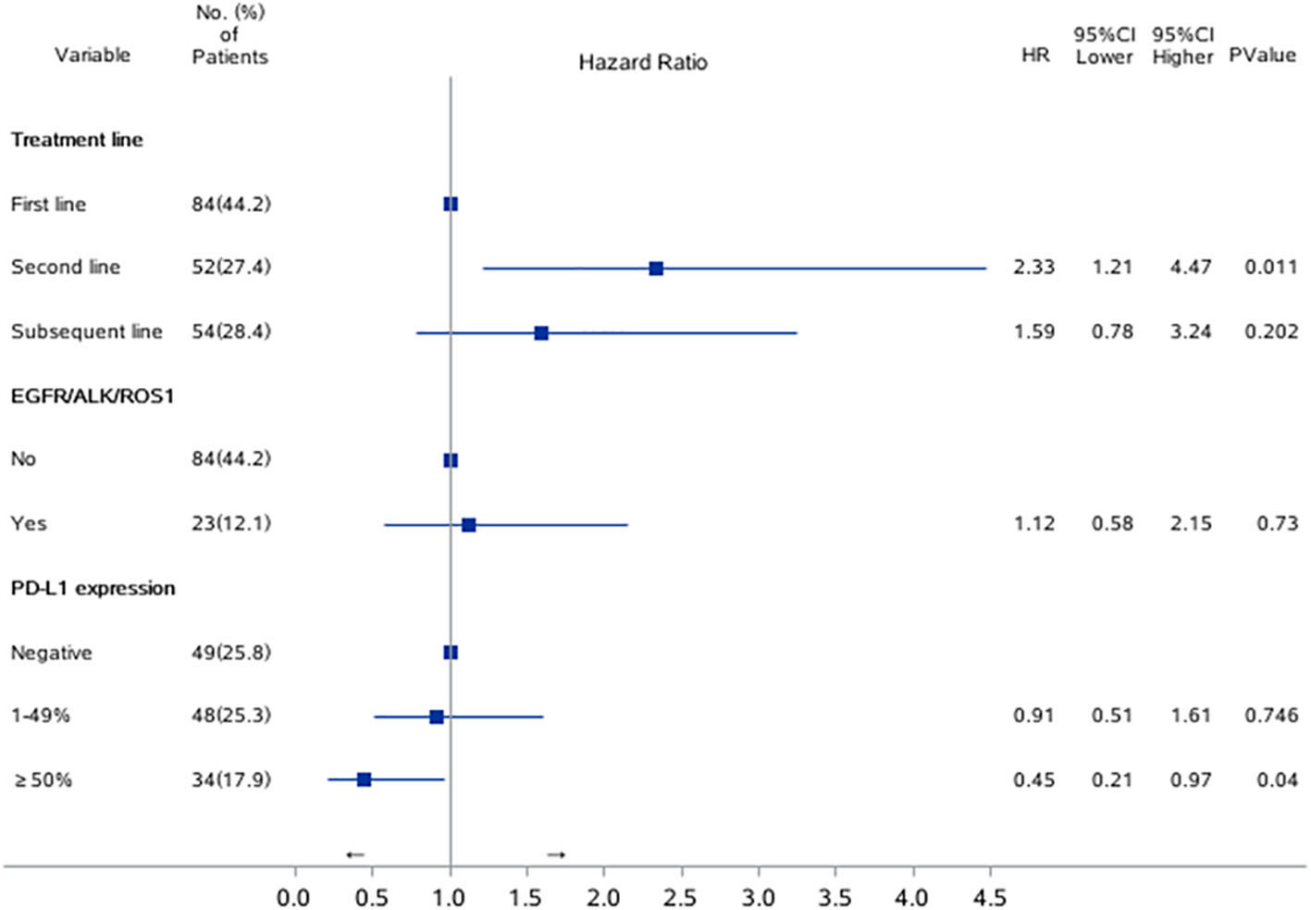
Risk factors for PFS1 in univariate and multivariate Cox regression model. Forest plots present hazard ratios and their confidence intervals (the horizontal lines) for PFS1 of the patients.

Univariable Cox proportional hazards regression analysis revealed that patients with EGFR/ALK/ROS1 mutation had a higher risk of ICI resistance (adjusted HR, 1.12; 95% CI, 0.58–2.15; P=0.0145). Multivariable Cox proportional hazards regression analysis revealed that second line immunotherapy (adjusted HR, 2.33; 95% CI, 1.21–4.47; P=0.011) was a significant risk factor associated with ICI resistance in lung cancer patients. PD-L1 expression of ≥50% was associated with a lower risk of ICI resistance (adjusted HR, 0.45; 95% CI, 0.21–0.97; P=0.04). (**Table 2**; **Figure 2**)

**Table 2:** Univariate and multivariate analysis of PFS1.

characteristics	mPFS1(95%CI)	p value	HR (95%CI)	p Value
Age				
≤65	7.29 (5.72, 10.51)	0.5423	—	—
>65	6.51 (5.45, 10.38)		—	—
Gender				
Male	7.16 (5.82, 10.25)	0.196	—	—
Female	5.62 (3.42, 10.51)		—	—
Smoking history				
Never	8.77 (5.62, 12.42)	0.7845	—	—
≤40 pack-years	6.64 (5.29, 9.66)		—	—
>40 pack-years	6.67 (5.32, 10.41)		—	—
Therapy				
Monotherapy	6.21 (4.83, 10.15)	0.2582	—	—
Combined chemotherapy	8.31 (5.82, 11.4)		—	—
Combined anti-angiogenic agents	3.81 (0.36, 10.51)		—	—
Treatment line				
First-line	9.66 (6.51, 12.91)	0.0303	ref	
Second-line	5.34 (3.25, 8.77)		2.33 (1.21,4.47)	0.011
Subsequent line	6.47 (5.22, 10.25)		1.59 (0.78,3.24)	0.202
Stage				
II/III	8.64 (5.68, 22.97)	0.1439	—	—
IV	6.64 (5.62, 9.66)		—	—
PS				
0	8.67 (5.75, 10.51)	0.152	—	—
1	6.67 (5.42, 10.38)		—	—
≥2	2.1 (0.41, 3.8)		—	—
EGFR/ALK/ROS1 mutation				
No	9.86 (5.72, 13.24)	0.0145	ref	
Yes	6.11 (2.2, 10.64)		1.12 (0.58,2.15)	0.73
PD-L1 expression				
Negative	5.72 (4.4, 9.86)	0.0039	ref	
1-49%	5.42 (4.27, 10.51)		0.91 (0.51,1.61)	0.746
≥50%	16.39 (10.93, 21.86)		0.45 (0.21,0.97)	0.04
Cancer type				
Adenocarcinoma	8.67 (5.52, 12.42)	0.2613	—	—
Squamous cell lung cancer	9.13 (6.11, 12.65)		—	—
Small cell lung cancer	5.68 (4.27, 6.01)		—	—
Other types	6.67 (5.22, 10.64)		—	—

**Figure 3:**
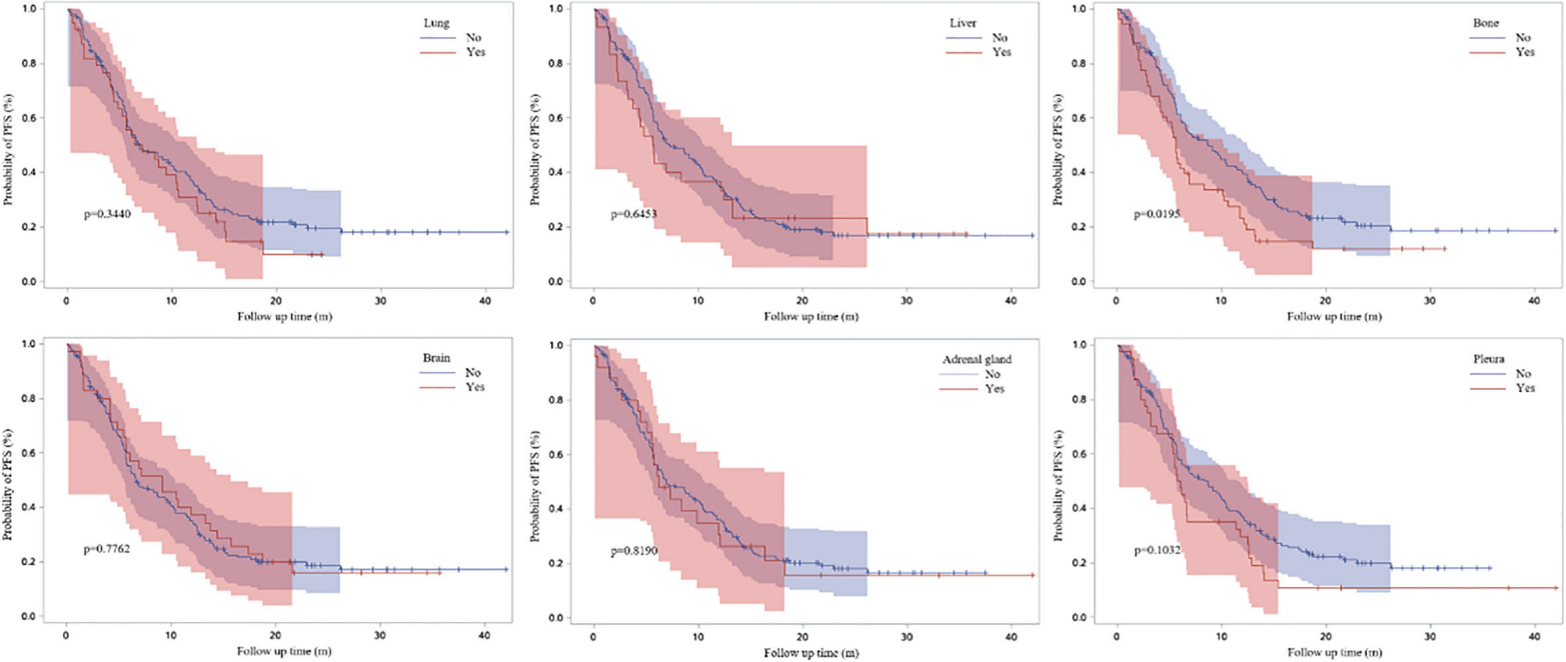
Survival curves based on Kaplan-Meier for different metastatic sites at baseline.

**Figure 3** demonstrated the Kaplan-Meier curves for different metastatic sites. We found that PFS1 time was significantly shorter for patients with bone metastasis at baseline (adjusted HR, 1.505; 95% CI, 1.036-2.186; P=0.0195).

### Clinical characteristics of ICI resistance

**Figure 4:**
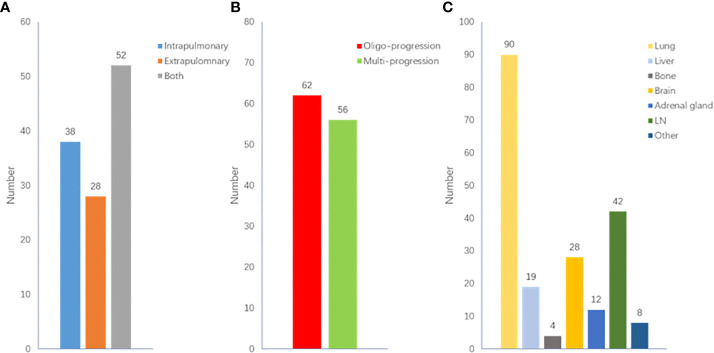
Clinical characteristics of ICI resistance. **(A)** Distribution of intra/extra-pulmonary progression; **(B)** Distribution of oligo/multi-progression; **(C)** Distribution of progression site.

When disease progression after the initial immunotherapy, 32.2% (N=38) of patients experienced intrapulmonary progression and 23.7% (N=28) experienced extrapulmonary progression *(* **Figure 4A** *)*. Among them, 52.5% (N=62) experienced oligo-progression (**Figure 4B**). Lung was the most common site suffering from tumor progression (**Figure 4C**).

### Different therapeutic regimens beyond ICI resistance

**Figure 5:**
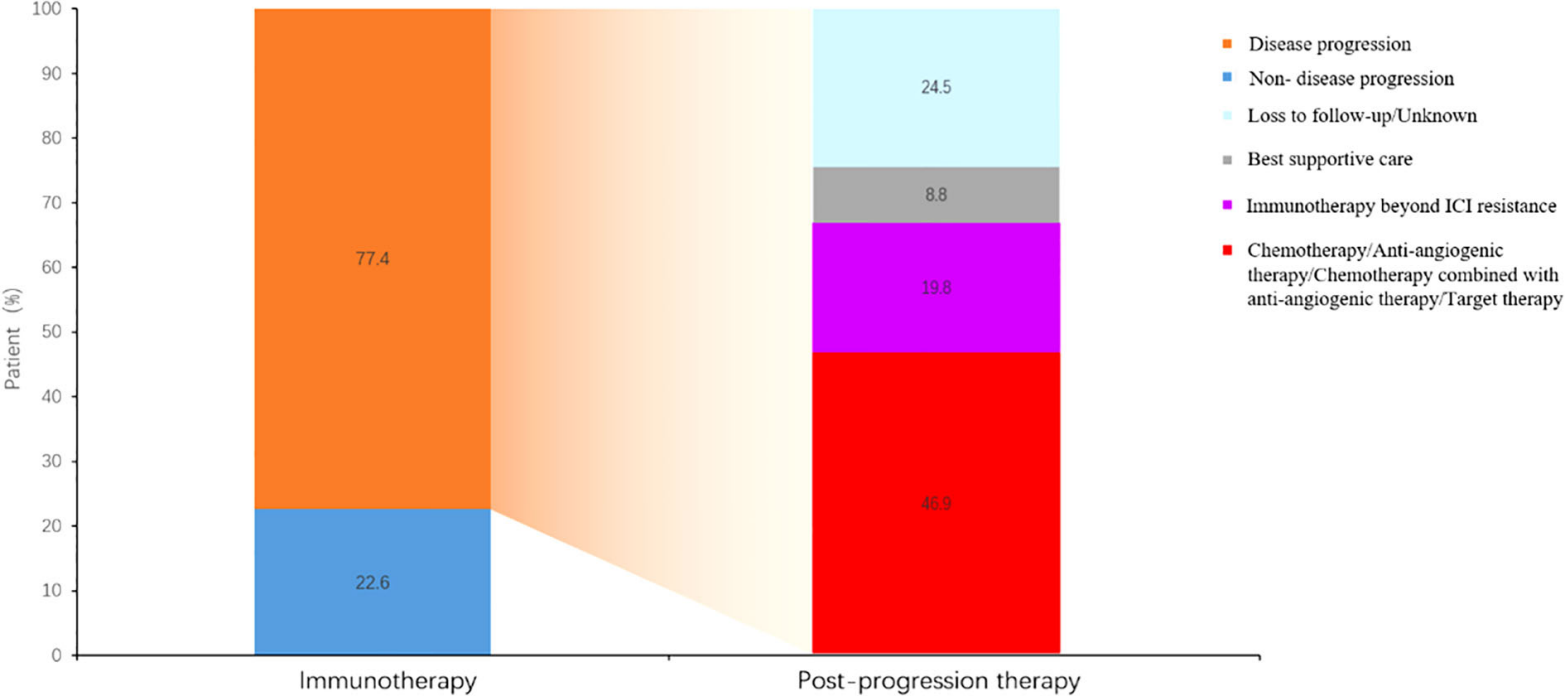
Therapeutic regimens beyond ICI resistance.

**Figure 6:**
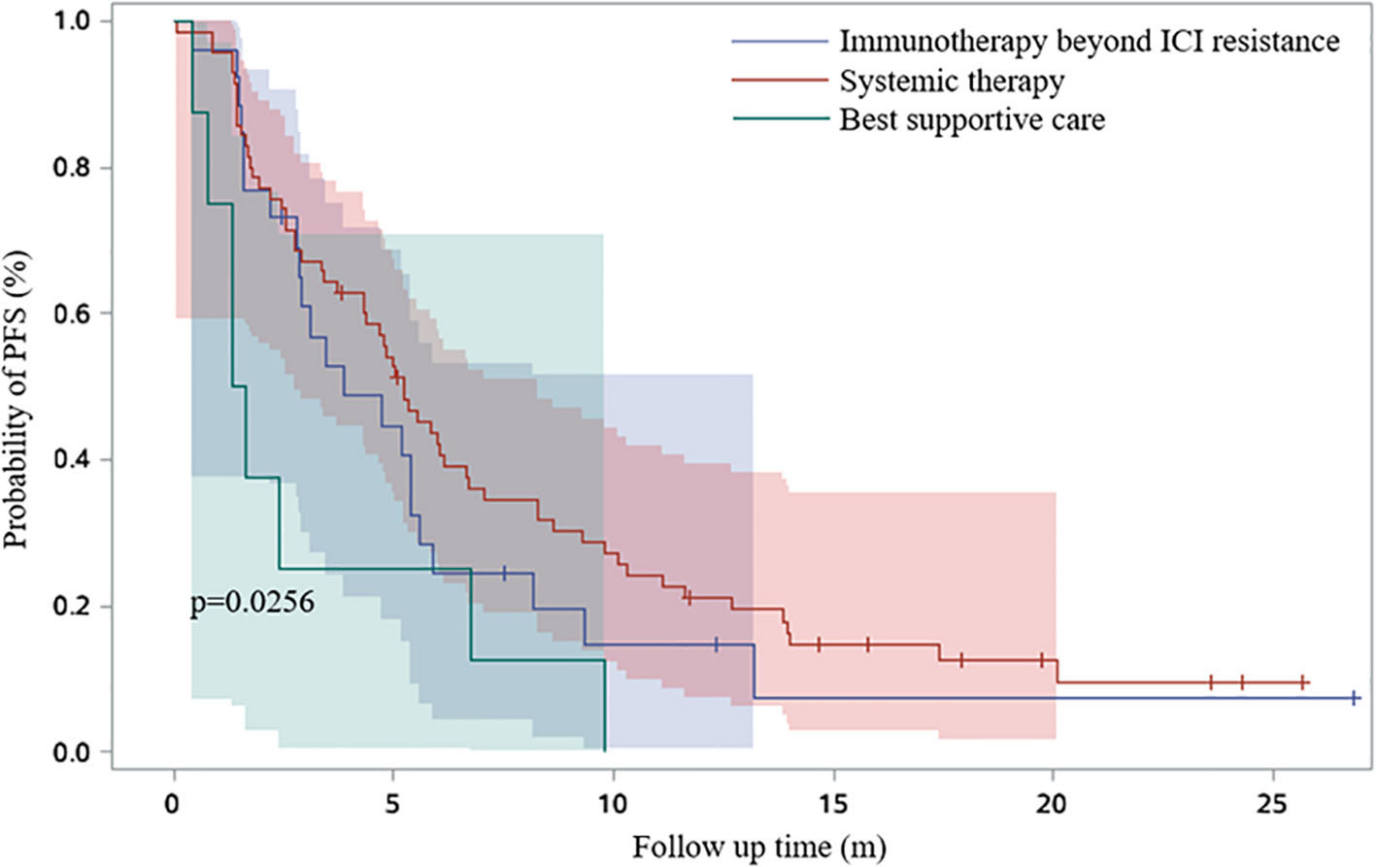
Survival curves based on Kaplan-Meier for different therapeutic regimens beyond ICI resistance.

**Figure 7:**
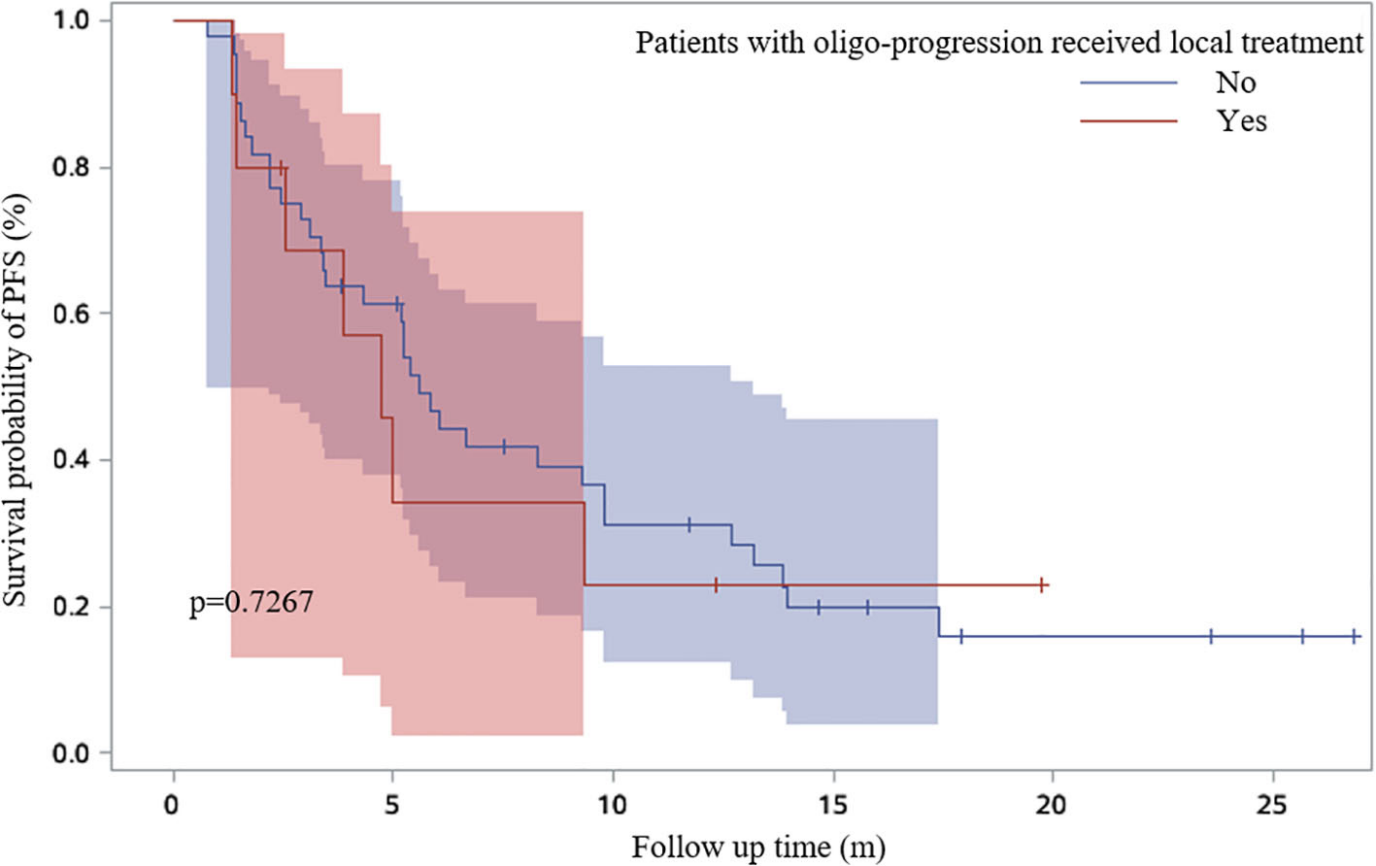
Survival curves based on Kaplan-Meier for whether or not patients with oligo-progression received local treatment.

19.8% patients received continued immunotherapy beyond ICI resistance (**Figure 5**). Systemic therapies including chemotherapy and anti-angiogenic therapy rather than continued immunotherapy beyond ICI resistance had significant effect on PFS2 time (P=0.0256) (**Figure 6**). 11/62 patients with oligo-progression received local treatment. Among them, 5 patients received cerebral radiotherapy, 5 patients received thoracic radiotherapy, and 1 patient received surgery for adrenal metastasis. Whether or not patients with oligo-progression received local treatment seemed to have no significant effect on PFS2 time (P=0.7267) (**Figure 7**).

### Different characteristics of ICI resistance and clinical outcomes

**Figure 8:**
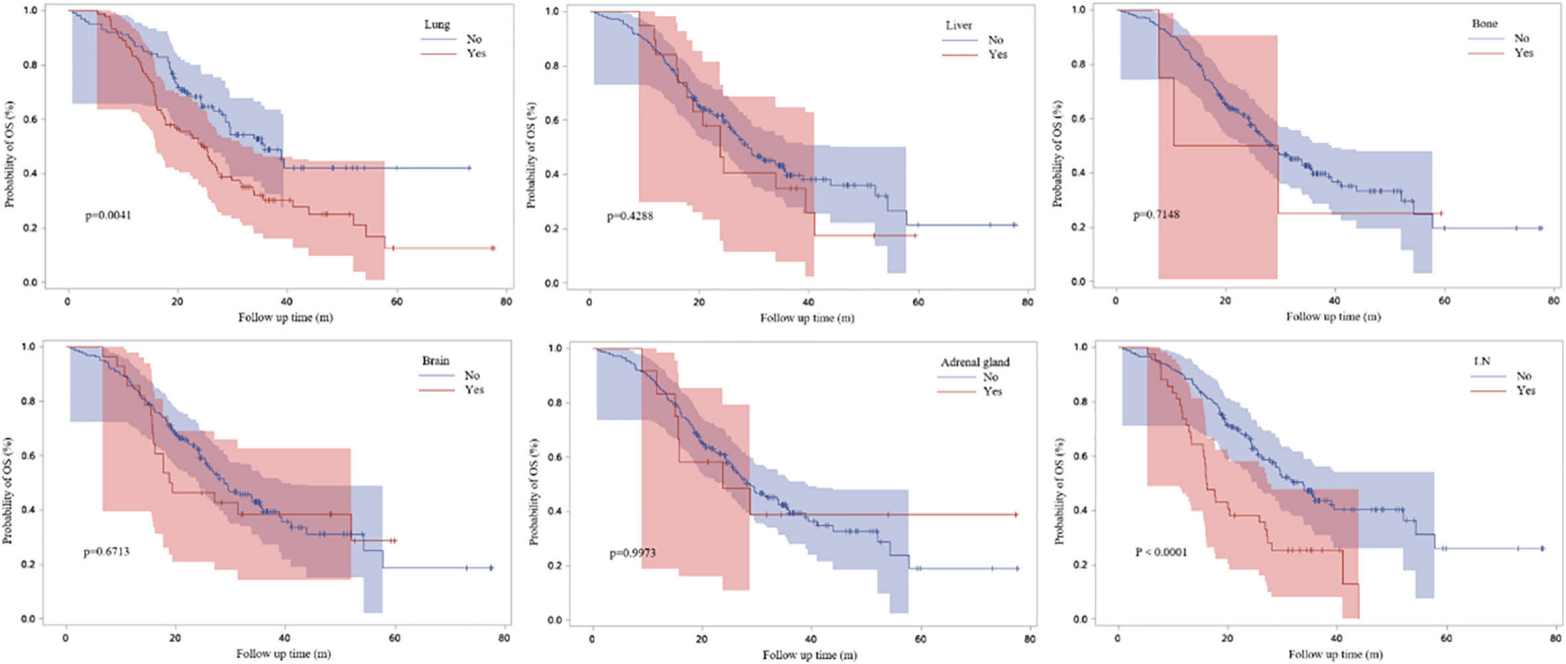
Survival curves based on Kaplan-Meier for different progressive sites.

**Figure 9:**
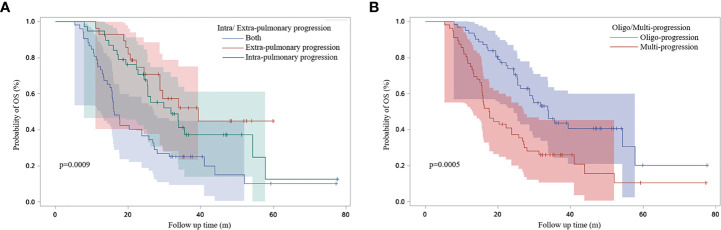
Survival curves based on Kaplan-Meier for different characteristics of ICI resistance. **(A)** Survival curves for patients with intra/extra-pulmonary progression; **(B)** Survival curves for patients with oligo/multi-progression.

Patients with lung or lymph nodes progression when ICI resistance had poor OS time (P=0.0041 and P<0.0001) (**Figure 8**). Patients with extrapulmonary progression and oligo-progression after initial exposure to immunotherapy had longer OS time than those with intrapulmonary progression and multi-progression (**Figures 9A, B**).

### Potential prognostic factors for ICI resistance

**Figure 10:**
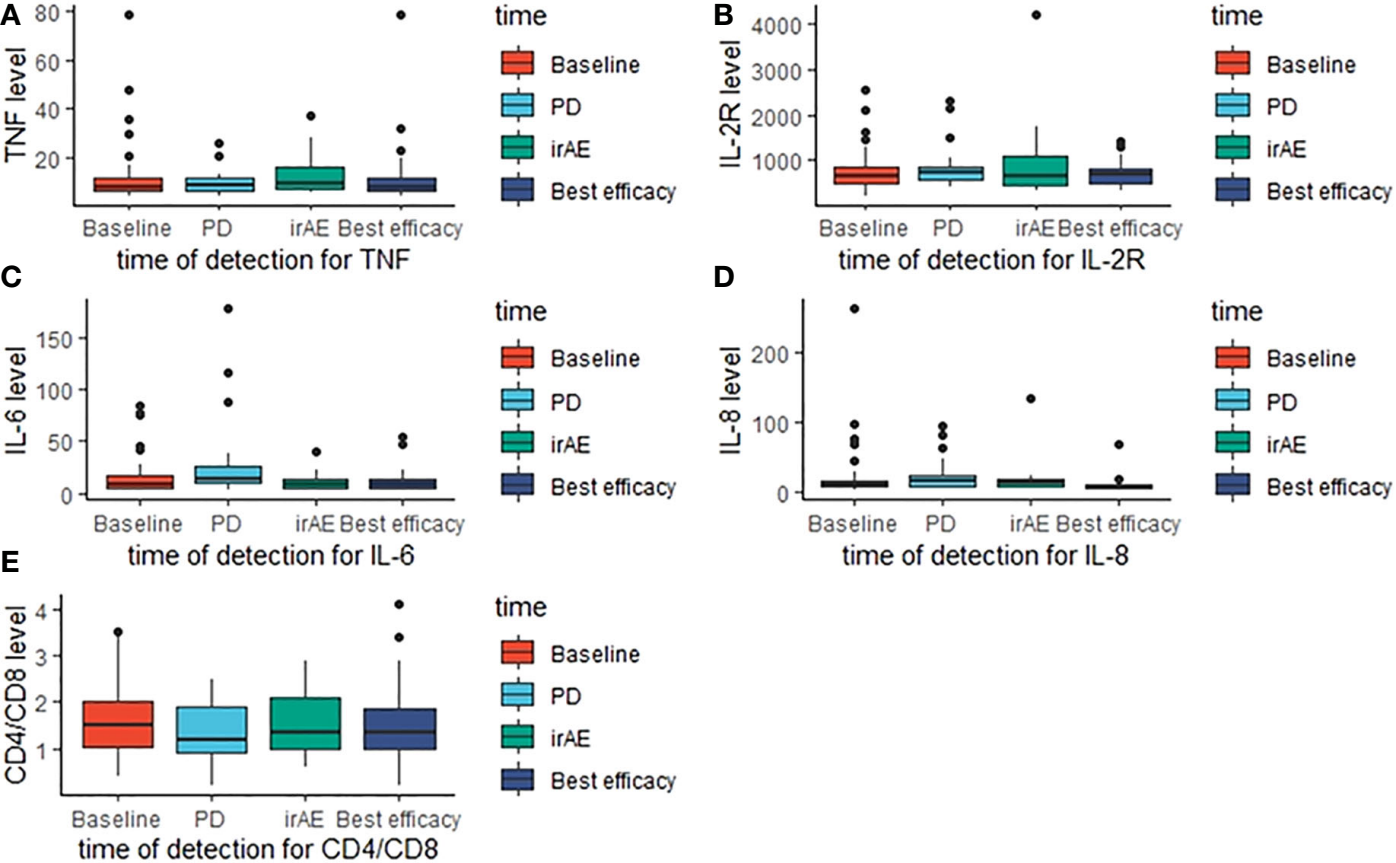
Cytokines levels at different detection timepoint. The boxplots present the distribution of cytokines levels at baseline, disease progression, irAE occurrence and best efficacy approached. **(A)** TNF levels; **(B)** IL-2R levels; **(C)** IL-6 levels; **(D)** IL-8 levels; **(E)** CD4/CD8 levels.

**Figure 11:**
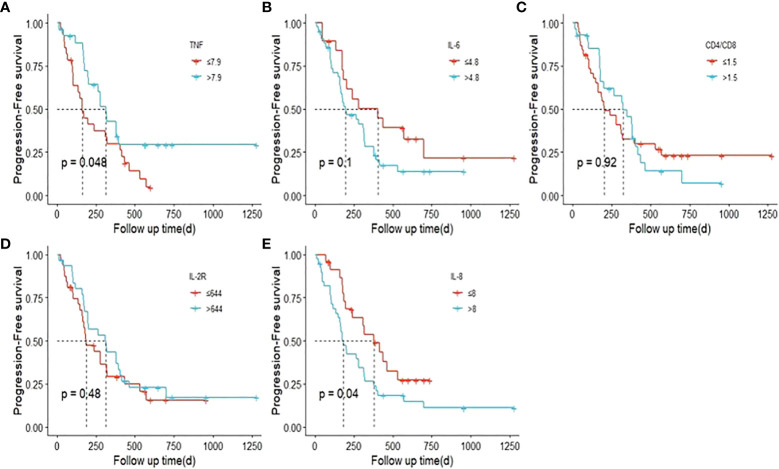
Survival curves based on Kaplan-Meier for different plasma cytokines. P values were calculated based on log-rank test. (PFS1). **(A)** TNF; **(B)** IL-6; **(C)** CD4/CD8; **(D)**. IL-2R; **(E)** IL-8.

Part of our patients had plasma cytokine data at different timepoint, including baseline, disease progression, occurrence of irAE and best efficacy. Based on the available data, we evaluated the potential prognostic value of plasma cytokines for the outcomes of ICI treatment. **Figures 10A-E** demonstrated the cytokine levels at different timepoint. When disease progression during the initial ICI treatment, levels of TNF, IL-6 and IL-8 were significantly elevated (**Table 3**). **Figures 11A-E** showed survival curves based on Kaplan-Meier for different plasma cytokines. Area under the ROC Curve of TNF is less than 0.5 (0.42), so we selected median value 7.9 pg/mL as the cutoff for TNF. Plasma TNF level higher than 7.9 pg/ml was significantly associated with longer PFS1 time (**Figure 11A**). We determined that 8 pg/mL is a clinically relevant threshold using ROC curve analysis. Plasma IL-8 level higher than 8 pg/mL was significantly associated with shorter PFS1 time (**Figure 11E**). These results suggested that TNF and IL-8 might serve as prognostic biomarkers for the PFS time of patients with advanced-stage lung cancer treated with ICI.

**Table 3:** Results of Wilcoxon test between cytokine levels at baseline and other detection timepoints.

Cytokine	Progress vs baseline		AE vs baseline		Best efficacy vs baseline	
	statistic	P value	statistic	P value	statistic	P value
TNF	177	0.0340	20.5	0.8588	237.5	0.4388
IL-2R	198	0.0703	31	0.7598	363.5	0.2628
IL-6	208	0.0085	17	0.5536	277.5	0.9644
IL-8	208.5	0.0081	37	0.0960	185.5	0.495
CD4/CD8	80.5	0.08255	25.5	0.8783	135.5	0.2025

**Figure 12:**
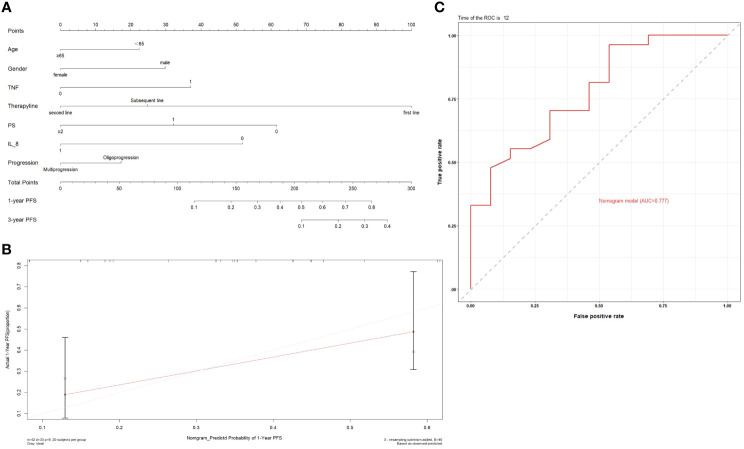
**(A)** Prognostic nomograms for predicting the PFS probabilities of patients; **(B)** Calibration curve of the prediction model; **(C)** ROC curve of the prediction model.

Next, we constructed a nomogram based on the plasma cytokines. The PFS nomogram included the following valuables: age, gender, TNF, IL-8, treatment line, PS and oligo/multi-progression (**Figure 12A**). One hundred was set as the maximum score of each variable; by adding up the total score and locating it on the bottom point scale, an estimated probability of survival could be easily determined. Calibration plot of the nomogram is displayed in **Figure 12B**. The C-index of the built nomogram to predict PFS (0.74; 95% CI, 0.65–0.82) demonstrated an optimal consistency between the nomogram prediction and the actual observation for lung cancer patients treated with ICI. The ROC curve indicated that the nomogram model has a good predictive ability in predicting the clinical outcomes of lung cancer patients receiving ICI treatment (AUC: 0.777 for 1‐year PFS) (**Figure 12C**).

## Discussion

ICIs targeting PD-1 and PD-L1 display notable clinical benefits for the treatment of advanced lung cancer. However, the limited benefit population, primary and acquired resistance and the lack of prognostic biomarkers have become the challenges related to the use of ICI treatment in clinical practice. Our retrospective study aimed at providing real world data on the clinical characteristics of ICI resistance, clarifying optimal treatment beyond ICI resistance and exploring potential prognostic factors.

In our study, 77.4% (N=147) of patients experienced disease progression during the initial ICI treatment. The median PFS1 was 6.9 months. We found that patients receiving ICI treatment as second-line therapy had higher risk of ICI resistance. EGFR/ALK/ROS1 mutation was also a risk factor of ICI resistance. This finding was in accordance with previous studies (7, 8)

It has been reported that bone is the most common site of metastasis from lung (9). 20-30% of patients already have bone metastasis when initially diagnosed with lung cancer (10). In our data, bone metastases were determined in 27.9% (N=53) of patients at diagnosis. Bone metastases usually features a short survival and poor prognosis in lung cancer patients (11, 12). In this novel data, we first found that PFS1 time was significantly shorter for patients with bone metastasis at baseline. In other words, patients with bone metastasis had poor prognosis during immunotherapy.

When ICI resistance, 32.2% (N=38) of patients developed intrapulmonary progression and 52.5% (N=62) developed oligo-progression. Lung was the most frequent site suffering from tumor progression. Previous study demonstrated that adding radiotherapy to pembrolizumab immunotherapy significantly increased responses and outcomes in patients with metastatic NSCLC (13). For patients with oligo-progression in our study, combination with local treatment did not lead to a significantly longer PFS2. The inconsistent results might be owing to the limited sample and different local treatment sites. 19.8% (N=29) of patients received continued immunotherapy beyond ICI resistance. Combination with chemotherapy (N=15, 51.7%) was the most common treatment, followed by continued immunotherapy with antiangiogenic therapy (N=9, 31.0%). Systemic therapies including chemotherapy and anti-angiogenic therapy rather than continued immunotherapy beyond ICI resistance had significant effect on PFS2 time.

We found that patients suffering from lung and lymph nodes progression after initial exposure to immunotherapy had poor prognosis. When resistant to immunotherapy, patients with extrapulmonary progression or oligo-progression had longer OS time than those with intrapulmonary or multi-progression.

Furthermore, we noticed that plasma cytokines could serve as potential prognostic factors of ICI resistance. Previous study has reported that elevated plasma IL-8 is associated with poor outcome of ICI treatment (14). In our study, we found that levels of TNF, IL-6 and IL-8 were significantly elevated when ICI resistance. Lower plasma TNF level and higher plasma IL-8 level seemed to be significantly associated with ICI resistance. The mechanistic basis of the prognostic roles of plasma cytokines requires further investigation.

We also established a nomogram to predict the clinical outcome of patients receiving ICI treatment. Age, gender, plasma cytokines, therapy line, PS and oligo/multi-progression were all factors contributed to the nomogram. Although we have conducted internal validation in our study, external validation should also be conducted in order to guarantee the repeatability and reliability of the established nomogram.

There were some limitations in our study. The retrospective study conducted in our single center might limited the generalizability of the results. Due to the retrospective nature of our study, the lack of complete data of plasma cytokines for all patients limited the conclusions regarding the roles of plasma cytokines played in ICI resistance.

## Conclusion

Patients with EGFR/ALK/ROS1 mutation and receiving ICI treatment as second-line therapy had higher risk of ICI resistance. Patients with bone metastasis at diagnosis had poor prognosis during immunotherapy. For those patients with oligo-progression after ICI resistance, combination with local treatment did not lead to a significantly longer PFS2 time. Systemic therapies including chemotherapy and anti-angiogenic therapy rather than continued immunotherapy beyond ICI resistance had significant effect on PFS2 time. TNF, IL-6 and IL-8 were associated with ICI resistance. The underlying mechanisms of resistance to immunotherapy need further clarified, which could guide drug development and lead to more precise therapy.

## Data availability statement

The raw data supporting the conclusions of this article will be made available by the authors, without undue reservation.

## Ethics statement

This study was approved by the ethics committee of Zhongshan Hospital, Fudan University (No. B2020-431R). The requirement for informed consent from the patients included in this study was waived due to the retrospective nature of the study, and any personal information from the data was removed beforehand.

## Author contributions

JBZ conducted this research and wrote the paper. XL and HZ performed the statistical analyses. ND, YZ, and XX provided the patient information. LG, JZ, and YS modified the paper. JH designed the research. All authors contributed to the article and approved the submitted version.
